# PAN-based activated carbon nanofiber/metal oxide composites for CO_2_ and CH_4_ adsorption: influence of metal oxide

**DOI:** 10.3906/kim-2012-37

**Published:** 2021-06-30

**Authors:** Çisem KIRBIYIKKURUKAVAK, Burak Zafer BÜYÜKBEKAR, Mustafa ERSÖZ

**Affiliations:** 1 Department of Chemical Engineering, Konya Technical University, Konya Turkey; 2 Department of Nanotechnology and Advanced Materials, Selcuk University Turkey; 3 Department of Chemistry, Selcuk University Turkey

**Keywords:** Activated carbon fibers, CO_2_and CH_4_adsorption, electrospinning, porous adsorbent

## Abstract

In the present study, we successfully prepared two different electrospun polyacrylonitrile (PAN) based-activated carbon nanofiber (ACNF) composites by incorporation of well-distributed Fe_2_O_3_ and Co_3_O_4_ nanoparticles (NPs). The influence of metal oxide on the structural, morphological, and textural properties of final composites was thoroughly investigated. The results showed that the morphological and textural properties could be easily tuned by changing the metal oxide NPs. Even though, the ACNF composites were not chemically activated by any activation agent, they presented relatively high surface areas (S_BET_) calculated by Brunauer–Emmett–Teller (BET) equation as 212.21 and 185.12 m^2^/g for ACNF/Fe_2_O_3_ and ACNF/Co_3_O_4_ composites, respectively. Furthermore, the ACNF composites were utilized as candidate adsorbents for CO_2_ and CH_4_ adsorption. The ACNF/Fe_2_O_3_ and ACNF/Co_3_O_4_ composites resulted the highest CO_2_ adsorption capacities of 1.502 and 2.166 mmol/g at 0 °C, respectively, whereas the highest CH_4_ adsorption capacities were obtained to be 0.516 and 0.661 mmol/g at 0 °C by ACNF/Fe_2_O_3_ and ACNF/Co_3_O_4_ composites, respectively. The isosteric heats calculated lower than 80 kJ/mol showed that the adsorption processes of CO_2_ and CH_4_ were mainly dominated by physical adsorption for both ACNF composites. Our findings indicated that ACNF-metal oxide composites are useful materials for designing of CO_2_ and CH_4_ adsorption systems.

## 1. Introduction

The anthropogenic greenhouse gasses concentrations have been reported to reach yet another high by the World Meteorological Organization (WMO) Greenhouse Gas Bulletin in 20191 WMO Greenhouse Gas Bulletin 2019.1. The two most long-lived greenhouse gasses are carbon dioxide (CO_2_) and methane (CH_4_). These gasses could remain in atmosphere and oceans for centuries. The latest analysis showed that the globally averaged concentrations of CO_2_ and CH_4_ reached 407.8 ppm and 1869.0 ppb, respectively. The source of these greenhouse gases emissions are mostly related to human activities such as agriculture, consumption fossil fuels, transportation and landfills [1,2]. To reduce the greenhouse gases emissions into the atmosphere, several techniques have been reported e.g. thermal oxidation, membrane separation, biofiltration, absorption, and adsorption [3–6]. It is well-known that adsorption is considered to be the most useful technique, especially for low concentration gas pollution. To date, different types of materials have been studied for efficient gas adsorption, including zeolites, metal–organic frameworks (MOFs), clays, silica, polymers, or carbonaceous materials [7]. Among all, carbonaceous materials offer many advantages over other adsorbents due to their low-cost and easy preparation, abundancy, selectivity, and nontoxicity. Additionally, their properties are more tuneable than other adsorbents [8]. Therefore, in any kind of adsorption processes, carbonaceous materials are most widely used adsorbents.

Carbonaceous materials have been gained a growing attention to apply in different research areas including electronics, tissue engineering, environmental processes, food processing, catalyst and so on [9,10]. Because, they have excellent structural and mechanical properties like low density, high porosity and large surface area, which help to use them in large scale applications. According to the application area, there are different types of carbonaceous materials. Among the different types, activated carbon nanofibers (ACNFs) are thought to be the most useful type, since they possess superior well-developed porous materials with uniform micropore size distributions over the other carbonaceous materials, such as granular and powder activated carbons or carbon fibers [12]. Due to their small-size characteristics, ACNFs show unique properties including large surface area, high adsorption capacity, and controllable surface functional groups [13,13]. All of these properties make ACNFs excellent candidate for adsorbents. To date, several research groups have reported attractive studies on the highly efficient adsorption capacity of ACNFs for the removal of formaldehyde, toxic dyes, indoor CO_2_, heavy metals, and so on [14–17]. However, most of these reports have generally focused on the development of surface properties by modification of activation procedure, thus there is limited studies whether improvement of adsorption capacities of ACNFs could be achieved by the preparation of their composites with different components. According to reports, the preparation of ACNFs composites with metal oxides enhances the gas adsorption capacities of the resulting adsorbent [18]. The presence of metal oxides in ACNFs composites not only shows a positive effect of on surface area and pore size distribution due to their catalytic activity during the carbonization process, but also their basic sites could increase the gas adsorption capacity by interacting with acidic gas molecules like CO_2 _[19–21]. Therefore, we discuss the fabrication of two different ACNF/metal oxide composites as candidate adsorbents for CO_2_ and CH_4_ adsorption. 

For the fabrication of functional nanofibers, it has been reported that the electrospinning is one of the most largely used technique [22]. Polyacrylonitrile (PAN) has been most utilized carbon source because of highly carbon yield, low cost, and mechanical strength of resulted ACNFs in electrospinning process. Many studies support that the PAN-derived CNFs/metal oxide composites with enhanced physical and structural properties could be prepared by electrospinning [23–25]. Considering these advantages, we aim to fabricate two different PAN based-ACNF composites by incorporation of well-distributed Fe_2_O_3_ and Co_3_O_4_ nanoparticles (NPs) for the adsorption of CO_2_ and CH_4_. Fe_2_O_3_, and Co_3_O_4_ are the most common investigated metal oxide semiconductor materials and they are n-type and p-type metal oxide semiconductors, respectively. According to studies reported, these are good candidates for gas adsorption process since Fe_2_O_3_ has variable oxidation state [26], while Co_3_O_4_can absorb more oxygen on the surface than on other p-type absorbents [27]. Here, the ACNF composites were fabricate by electrospinning PAN solutions containing iron and cobalt precursor salt, followed by a thermal treatment. The structural and textural properties of electrospun ACNF/Fe_2_O_3_ and ACNF/Co_3_O_4_ composites fabricated were characterized to determine their potential use in gas adsorption processes. The effect of the different metal oxide types on the surface basicity, thereby, their adsorption capacities for CO_2_ and CH_4_ was discussed in detail. With this report, it could be said that the proposed approach is easy and effective, since it suggests a useful method to produce highly efficient gas adsorbents for future commercial applications. 

## 2. Experimental 

### 2.1. Materials

PAN (M_w_= 160,000 g mol^−1^), N,N-dimethylformamide, (DMF, anhydrous, 99.8%), iron(III) chloride hexahydrate (FeCl_3_.6H_2_O, 97.0%), sodium hydroxide (NaOH, anhydrous, 98.0%), cobalt(II) chloride hexahydrate (CoCl_2_.6H_2_O, 98.0%), sodium carbonate (Na_2_CO_3_, anhydrous, 99.9%), and absolute ethanol were purchased from Sigma–Aldrich Corp. and reagents were used as received.

### 2.2. Synthesis of metal oxide NPs

Both metal oxide NPs were synthesized by co-precipitation method as followed [19, 28]. 

**Synthesis of Fe** **_2_** **O** **_3_** **NPs:** 4 g of FeCl_3_.6H_2_O was used as precursor and dissolved in 25 mL of deionized (DI) water followed by stirring for 30 min at room temperature. Then, 25 mL of 0.7 mol/L NaOH solution was added to this solution drop by drop. The resulted solution was stirred magnetically for 2 h at 60 °C. The precipitates were centrifuged at 10,000 rpm followed by washing three times with DI water and ethanol, respectively.

**Synthesis of Co** **_3_** **O** **_4_** **NPs:** 5 g of CoCl_2_.6H_2_O was dissolved in 35 mL of DI water and stirred for 30 min at room temperature. After that, 40 mL of 1 mol/L Na_2_CO_3_ solution was dropped into the solution. The mixture was stirred for 5 h at 60 °C. After that, the collection and washing procedures of precipitates were performed as same as procedure mentioned above. It should be note that the precipitates were Fe(OH)_3_ and Co(OH)_2_ before the calcination process applied in preparation of ACNF/metal oxide composites given below.

### 2.3. Preparation of electrospun ACNF/metal oxide composites

**Figure 1:**
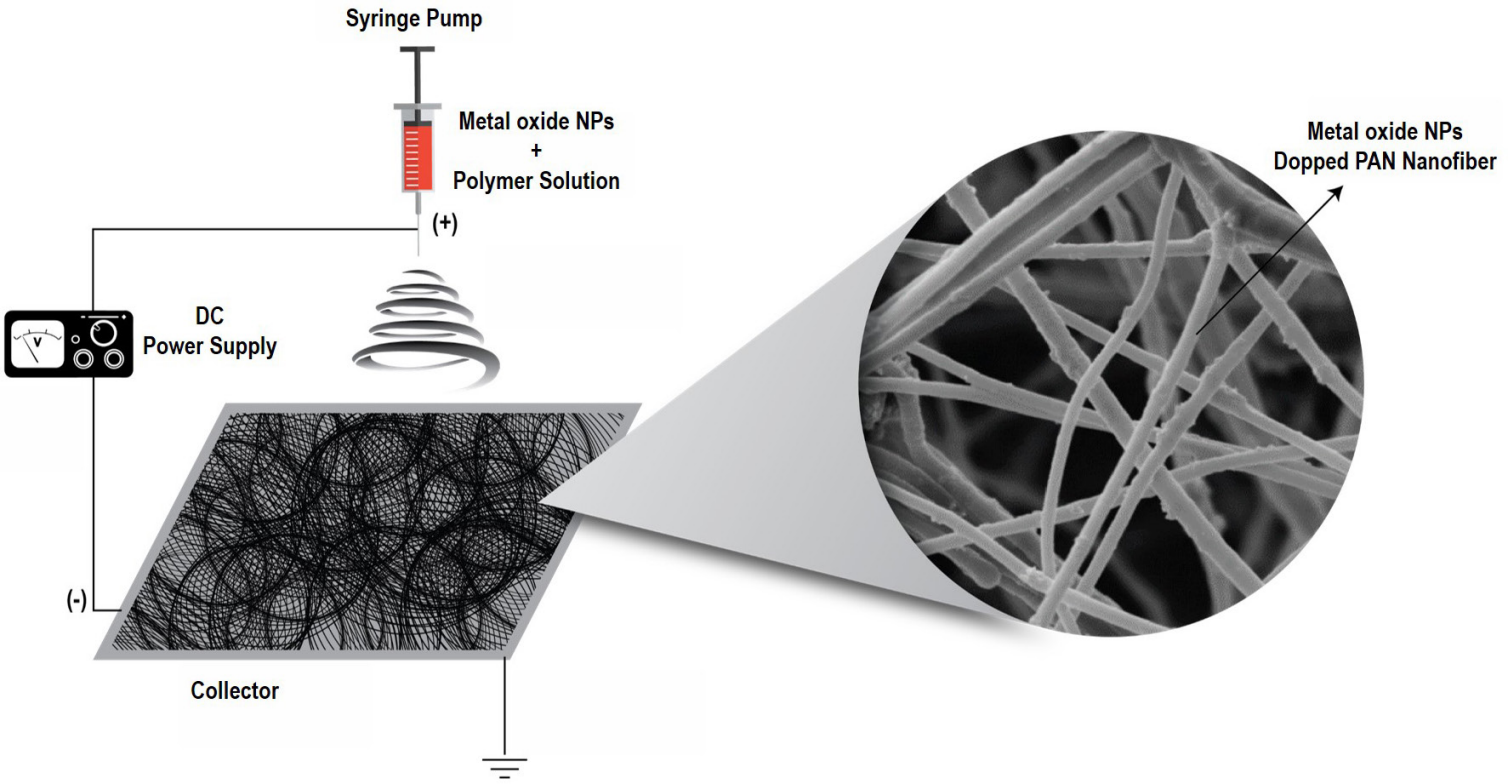
The preparation method of ACNF composites.

**Figure 2:**
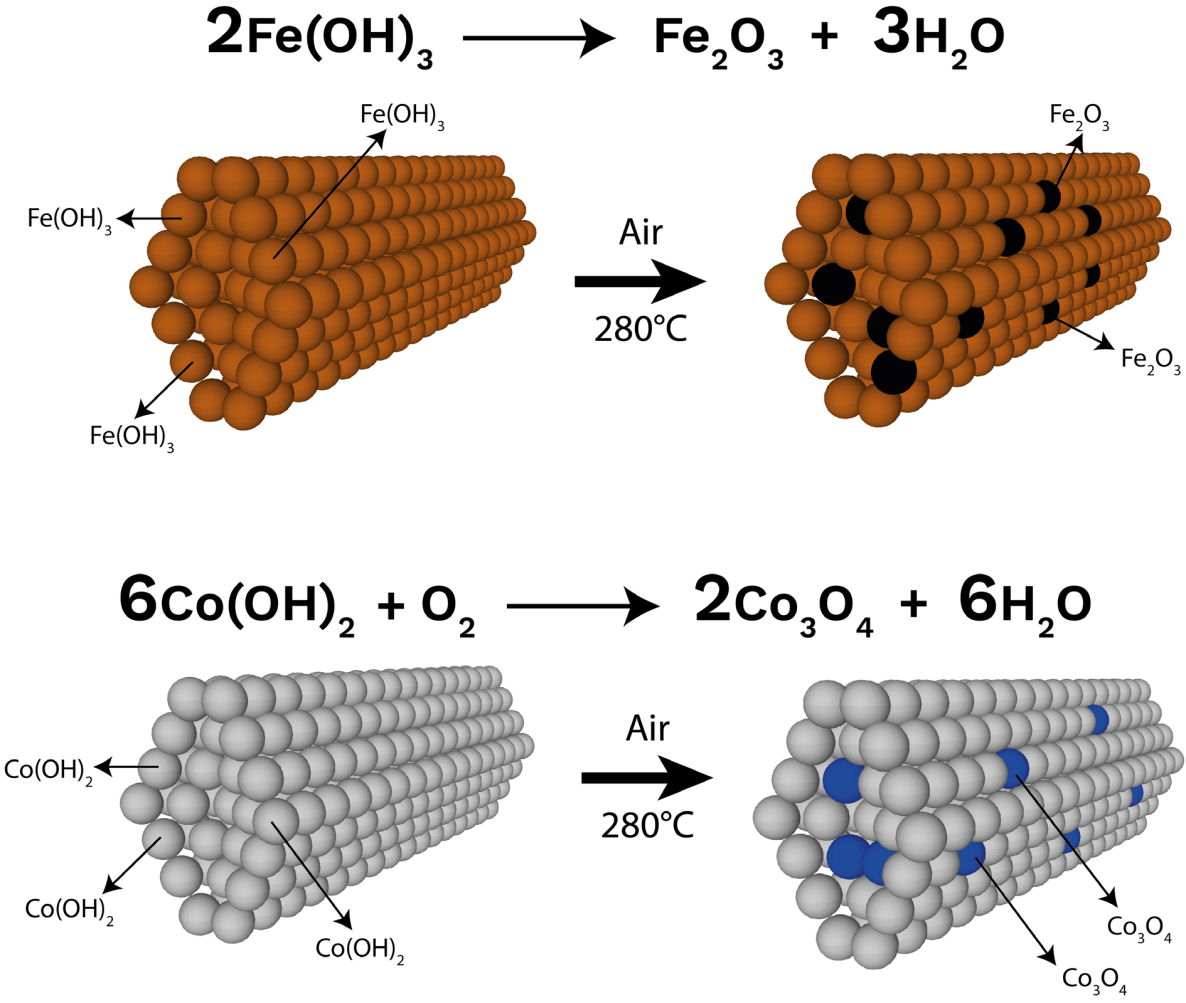
The schematically illustration of the ACNF composites and the reactions

ACNF/metal oxide composites were prepared via electrospinning of PAN/NPs solutions with PAN and metal oxide NPs in DMF at a weight ratio (PAN: DMF: metal oxide NPs) of 0.9: 9.0: 0.01 g. Before the electrospinning, both mixtures were stirred for 3 h at 60 °C to provide homogeneity. The schematically illustration of experimental procedure is given in Figure 1. Additionally, the schematically illustration of the ACNF composites and the reactions were given in Figure 2. PAN/NPs solutions prepared were filled into a 10 mL syringe with a stainless needle and then, placed in a syringe pump (KD Scientific). The tip was connected to the high-voltage power supply (Spelmann SL30). A grounded collector with a piece of aluminium foil was used to collect the nanofibers. The electrospinning parameters were applied as follows: the distance between the tip and nanofiber collector is 20 cm, the voltage applied is 30 kV and the pumping rate is 0.1 mL/min. After completion of electrospinning, the nanofibers were kept in air atmosphere for overnight, followed by peeling off from the aluminium foil. The peeled nanofibers were placed into a quartz boat for the calcination process in a tubular oven. Firstly, both nanofibers were heated to 280 °C with a heating rate of 5 °C/min in atmosphere and kept at final temperature for 1 h. After that, the stabilized Fe_2_O_3_ and Co_3_O_4_ added nanofibers were heated to 800 °C and 700 °C for thermal activation, respectively, with the same heating rate under nitrogen flow. The nanofibers were kept at final temperature for 1 h, then cooled to room temperature. 

### 2.4. Characterization of ACNF/metal oxide composites

The surface morphology and elemental analyses of ACNF/Fe_2_O_3_ and ACNF/Co_3_O_4_ composites were performed by scanning electron microscopy (SEM, Zeiss Evo) with energy dispersive X-ray analysis (EDX). For the structural analyses, X-ray diffraction (XRD) patterns was recorded by a Bruker D8 Advance diffractometer using CuKα radiation (λ = 0.15406 nm). Fourier transform infrared spectroscopy (FTIR) analysis was performed by Bruker Vertex 70 apparatus. Thermal gravimetric analysis (TGA) was used to identify the decomposition properties of ACNF composites. The surface area and pore characteristics were determined by N_2_ adsorption/desorption by a Micromeritics TriStar II adsorption apparatus. After the outgassing of certain amount of ACNF composites at 300 °C under vacuum for 24 h, adsorption/desorption experiment were conducted at –196 °C a relative pressure (p/p°) 0–1 and then back. The specific surface area (S_BET_) and micropore volume (V_micro_), total pore volume (V_total_) were calculated by Brunauer–Emmett–Teller (BET) equation and t-plot method, respectively. The average pore diameter and pore size distribution were determined by applying Barrett–Joyner–Halenda (BJH) equation. CO_2_ and CH_4_ adsorption-desorption measurements were also performed using a Micromeritics TriStar II adsorption apparatus at three different temperatures (0 °C, 25 °C, and 35 °C) up to 120 kPa. As mentioned above, certain amount of ACNF composites were outgassed at 300 °C before adsorption experiment. 

## 3. Results and discussion

**Figure 3:**
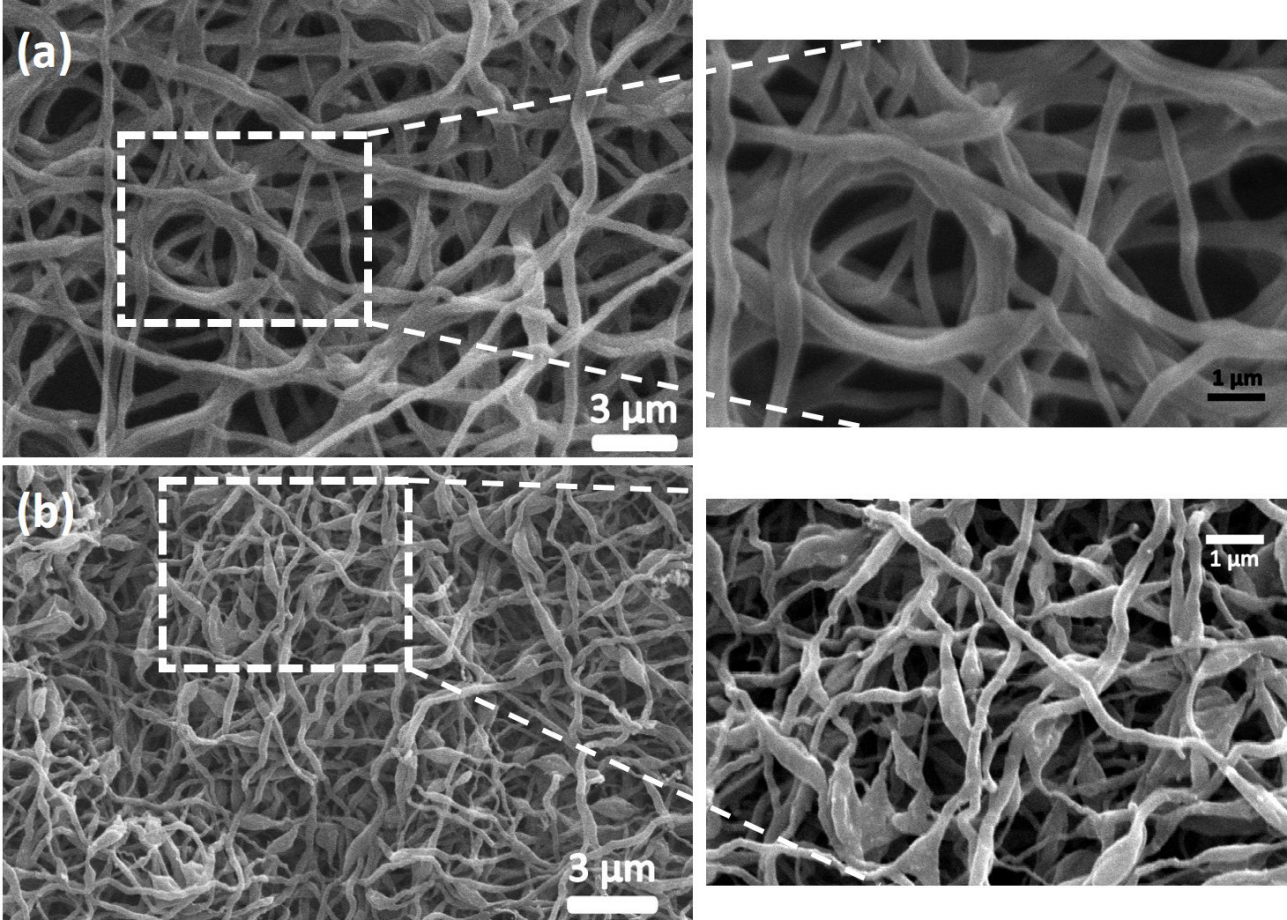
SEM images of PAN based (a) ACNF/Fe2O3 and (b) ACNF/Co3O4 composites.

**Figure 4:**
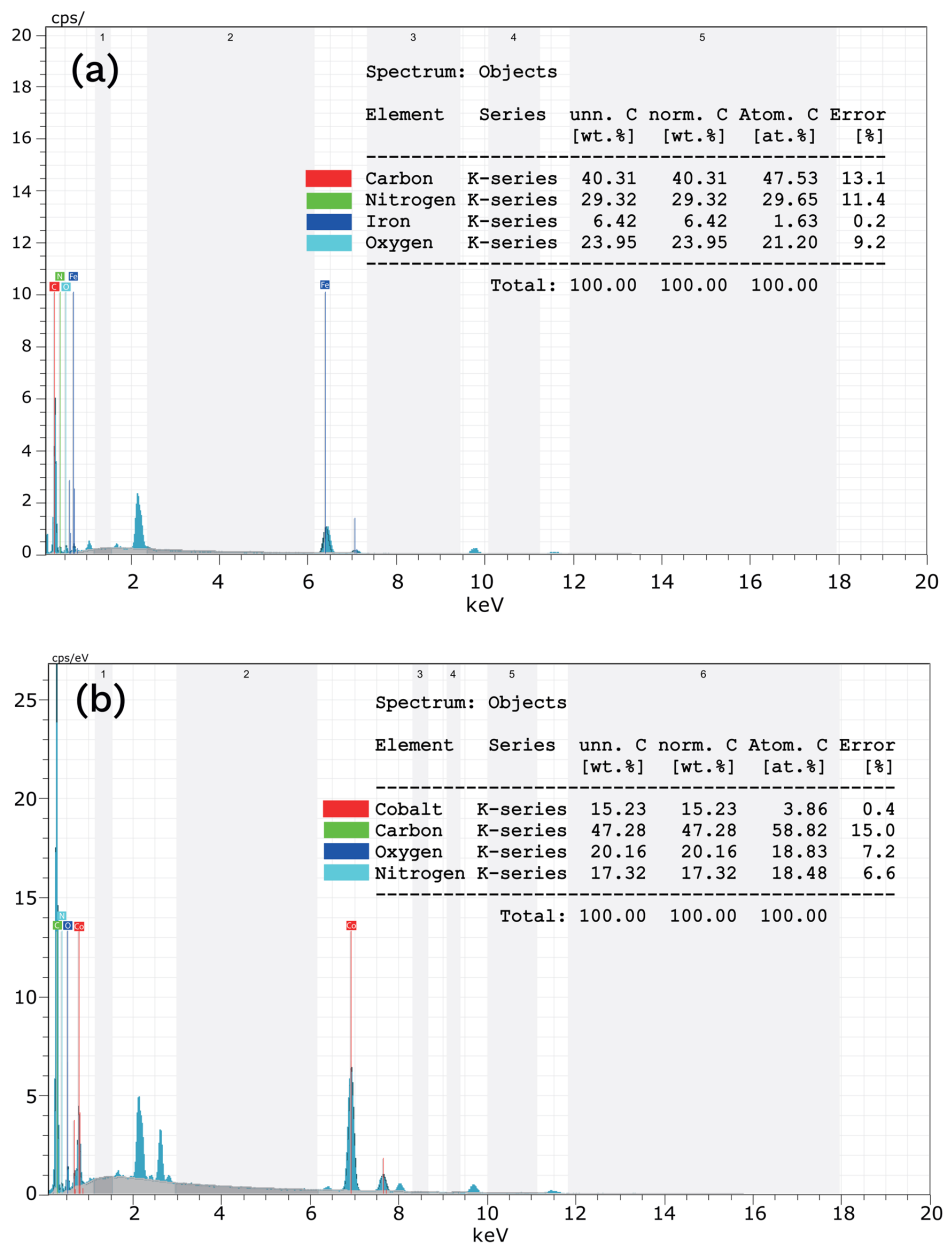
EDX analyses of (a) ACNF/Fe2O3 and (b) ACNF/Co3O4 composites.

To illuminate the morphological characteristics of adsorbents, Figure 3 (a) and (b) presents SEM images of ACNF/Fe_2_O_3_ and ACNF/Co_3_O_4_ composites, respectively. It could be said that both composites have continuous and long morphologies. As seen, ACNF/Fe_2_O_3_ composite shows a relatively thick cylindrical fibers with an average diameter of 160 nm ± 10 nm, whereas ACNF/Co_3_O_4_ composite reveals a rough surface and thinner fibers with an average diameter of 130 ± 15 nm. As seen, the rough surface of ACNF composites could be attributed to the presence of metal oxide NPs agglomerated [29]. From these images, both composites have highly porous surfaces, which could lead to increase adsorption capacity. The corresponding EDX analyses of ACNF/Fe_2_O_3_ and ACNF/Co_3_O_4_ composites are given in Figure 4 (a) and (b), respectively. As expected, the highest element is carbon with high nitrogen content in both composites’ composition. The presence of metal oxide NPs is proven, since the existence of iron and cobalt elements are also detected in ACNF/Fe_2_O_3_ and ACNF/Co_3_O_4_ composites, respectively, in addition to high oxygen content. 

**Figure 5:**
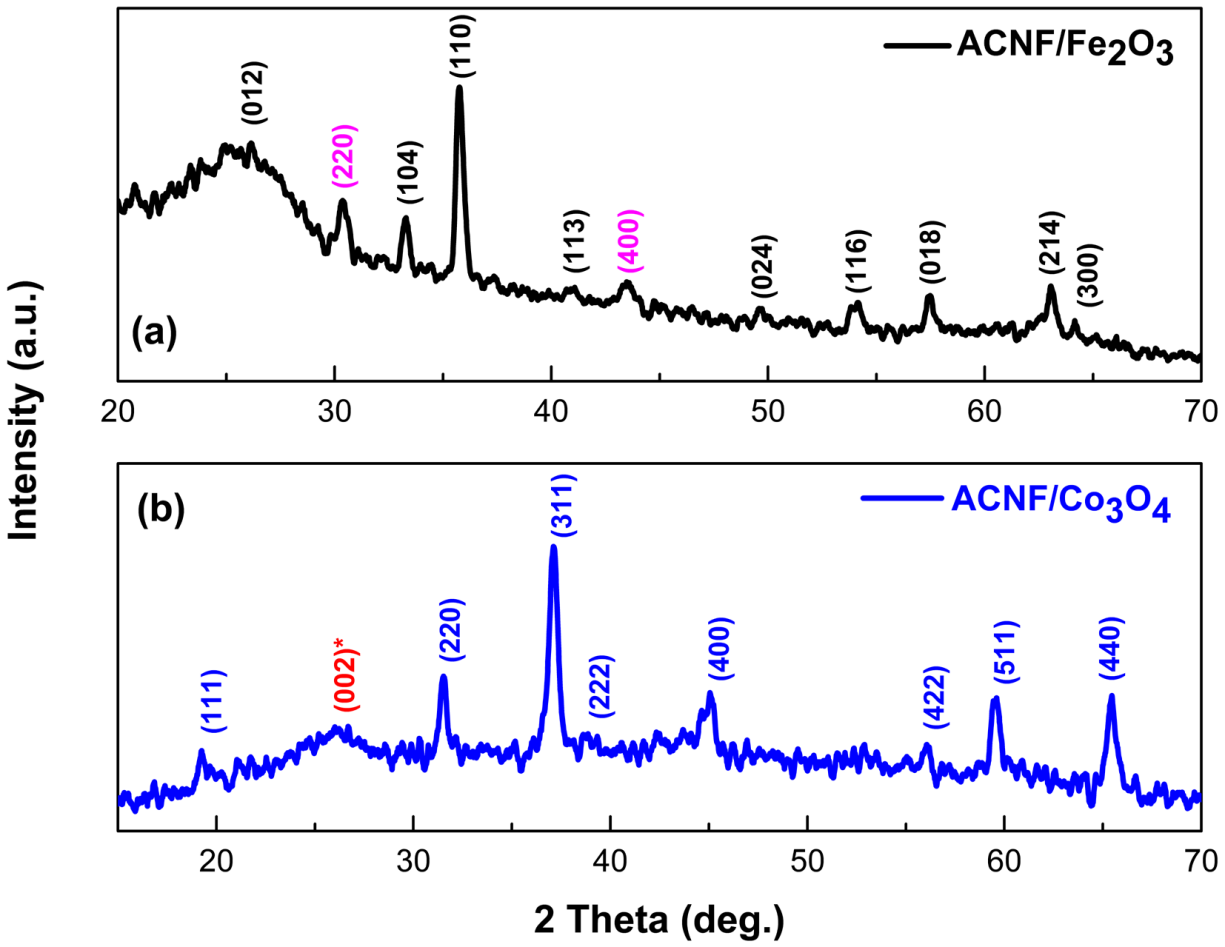
XRD patterns of the (a) ACNF/Fe2O3 and (b) ACNF/Co3O4 composites.

The XRD patterns of ACNF/Fe_2_O_3_ and ACNF/Co_3_O_4_ composites are shown in Figure 5 (a) and (b), respectively. As seen, both patterns present broad peaks at around 2θ = 25° corresponding to the reflection of (002) plane indicating the amorphous state of carbon fibers. In the pattern of ACNF/Fe_2_O_3_, several peaks corresponding to reflection of (012), (104), (110), (113), (024), (116), (018), (214), and (300) planes is assigned to the formation of α-Fe_2_O_3_ (hematite) (JCPDS File No. 24-0072). The weaker peaks corresponding to (220) and (400) planes could be attributed to the formation of γ-Fe_2_O_3_ as a secondary phase in composition (JCPDS File No. 39–1346) [31]. The XRD pattern of ACNF/Co_3_O_4_ composite shows eight sharp and strong diffraction peaks corresponding to the planes of (111), (220), (311), (222), (400), (422), (511), and (440), which presents a good matching with the standard JCPDS data of cubic Co_3_O_4_ NPs (JCPDS File No. 42-1467) [31]. The average particle size of Fe_2_O_3_ and Co_3_O_4_ NPs in ACNF composites were calculated from XRD patterns using Debye–Scherrer equation [32]. The average particle size calculated for Fe_2_O_3_ and Co_3_O_4_ NPs were 14.7 and 11.6 nm, respectively. 

**Figure 6:**
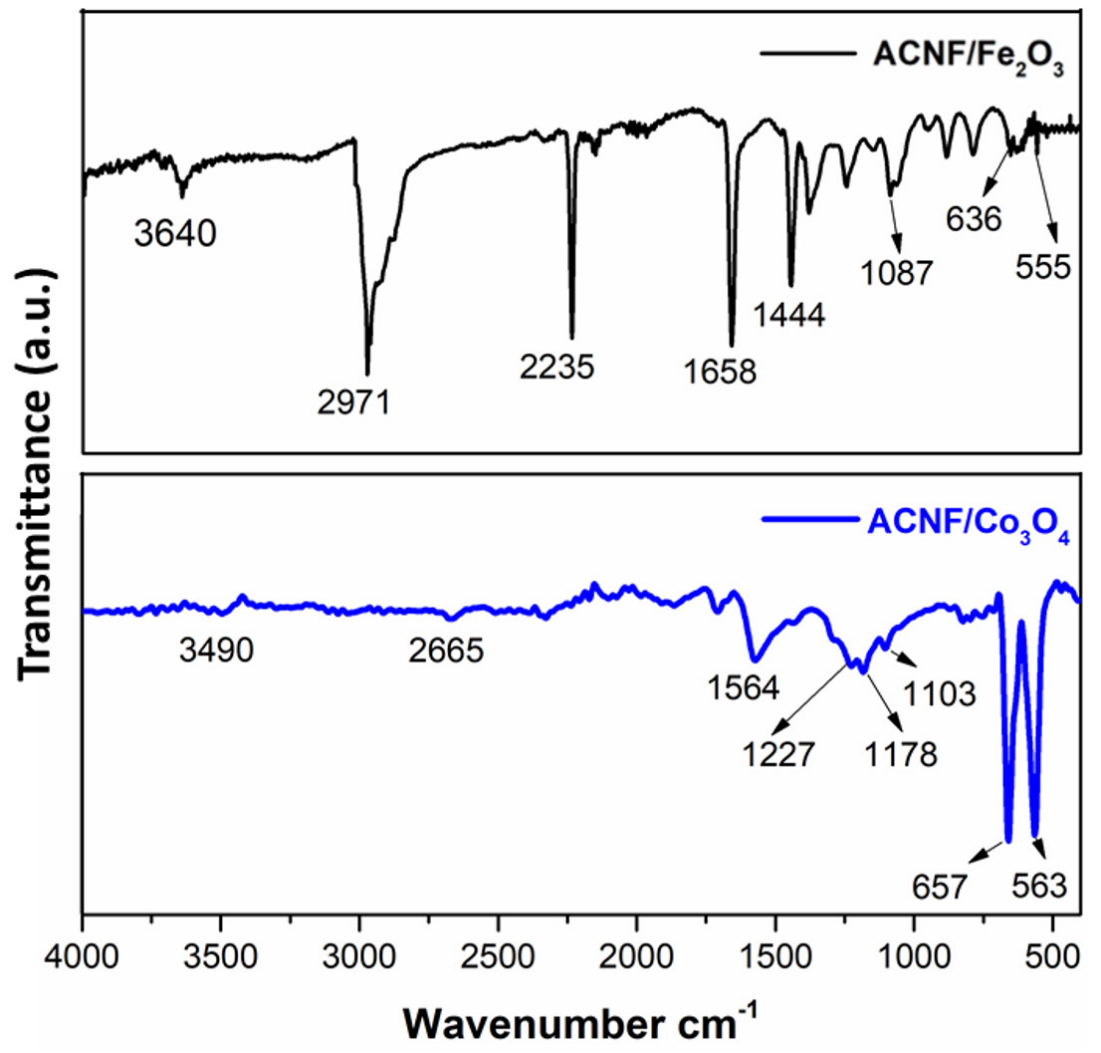
FTIR spectra of ACNF composites.

To provide better understanding on the structural properties of ACNF composites, FTIR analysis were performed and the results are seen in Figure 6. Both FTIR spectra showed peaks at approximately 3650 and 3500 cm^−1^ related to stretching vibration of O–H. In ACNF/Fe_2_O_3_ spectrum, the sharp and intense peaks at 2971 and 1444 cm^−1^ attributed to stretching and bending vibration of C–H, respectively, while the sharp peaks at 1658 and 1087 cm^−1^ related to stretching vibration of C=C bonds [33]. The sharp peak at 2235 cm^−1^ in ACNF/Fe_2_O_3_ spectrum could be related to stretching vibration of –CN bonds. The peaks at 636 and 555 cm^−1 ^accounted for the indication of Fe–O stretching vibration [35]. In ACNF/Co_3_O_4_ spectrum, the peaks at 2665 and 1103 cm^−1^ attributed to stretching and bending vibration of C–H, respectively, while the sharper peaks at around 1600 and 1200 cm^−1^ were assigned to the stretching vibration of C=C and C–O–H bonds, respectively [36]. The peaks at 657 and 563 cm^−1 ^were characteristic of Co–O vibrations [36]. According to the FTIR spectra, no additional peaks indicates that metal oxide nanoparticles successfully incorporated to nanofibers while electrospinning [37], which matches with the findings in SEM images. 

**Figure 7:**
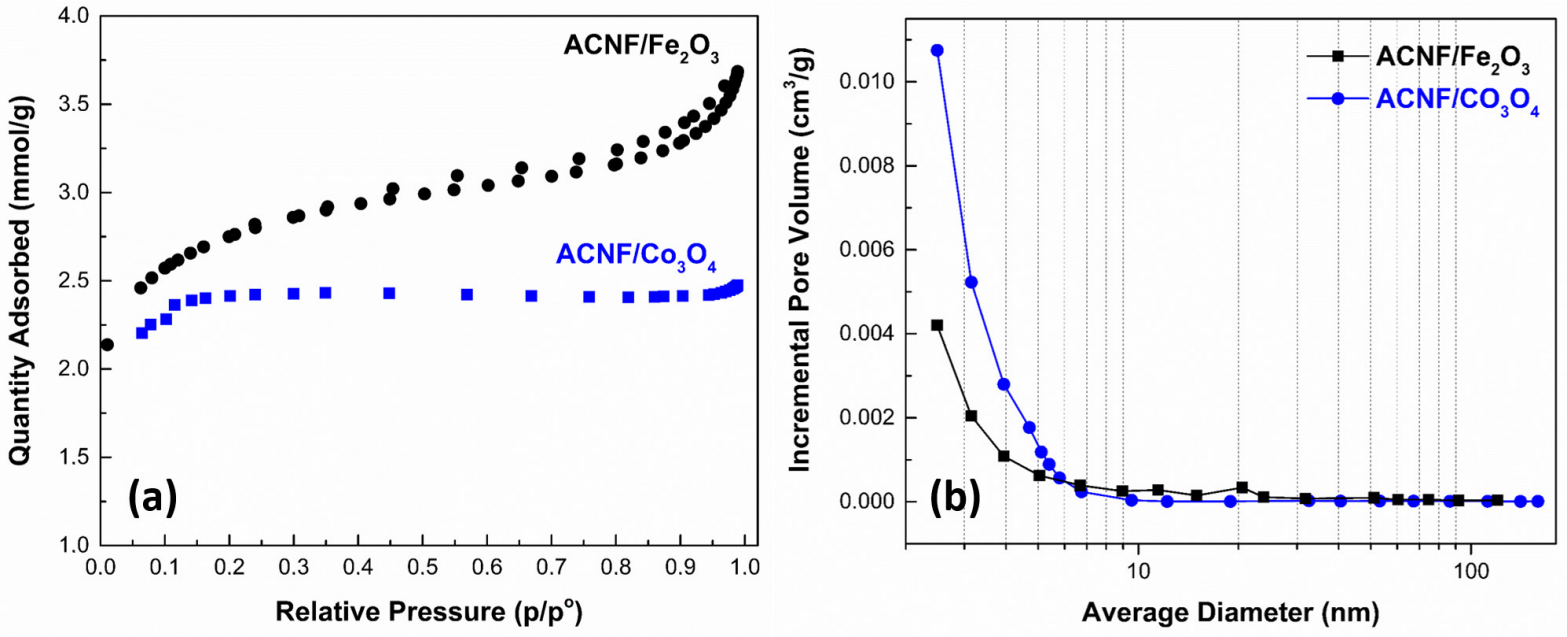
(a) N2 adsorption/desorption isotherms and (b) pore size distribution curves of ACNF composites.

Besides the presence of metal oxides, textural properties and pore system of an adsorbent are important parameters for gas adsorption studies. As well known, a higher surface area provides higher potential adsorption sites. The textural characteristics of ACNF composites includingS_BET_, V_micro_, V_total_, average pore diameter, and average pore width were investigated by N_2_ adsorption-desorption experiments and the results are seen in Table 1. Figure 7 (a) showed that the adsorption-desorption isotherms of both ACNF composites could be classified as the type I of IUPAC classification, which implies to microporous adsorbent texture. Even though, there are small hysteresis observed in both isotherms, this indicates that the composites show also mesoporous texture. S_BET_ values of ACNF/Fe_2_O_3_ and ACNF/Co_3_O_4_ composites were determined to be 212.21 and 185.12 m^2^/g, respectively. Since the ACNF composites were not chemically activated by any activation agent, the S_BET_ values are comparable with the surface areas of carbonized PAN nanofibers reported in literature [38]. Zhang et al. reported that the surface area of PAN-based carbonization nanofiber membranes prepared with different voltages applied changed from 61.4 to 21.2 m^2^/g [39]. In a different study, Kaerkitcha et al. prepared CNFs by electrospinning of PAN solution using single nozzle and they reported that the surface area of CNFs determined to be 164.7 m^2^/g [40]. Therefore, it can be said that these low S_BET_ values are expected and acceptable results for the conditions selected in this study. The ACNF/Fe_2_O_3_ showed a V_total_ of 0.125 cm^3^/g with a V_micro_ of 0.054 cm³/g, whereas The ACNF/Co_3_O_4_ showed a V_total_ of 0.085 cm^3^/g with a V_micro_ of 0.055 cm³/g. The ACNF/Co_3_O_4_ presented a V_total_, approximately twice that of ACNF/Fe_2_O_3_. With this, it could be said that the textural properties of both composites were quite effected depending on the presence of metal oxide. The pore size distribution curves of ACNF composites are presented in Figure 7 (b). As seen, the significant range of pore size distribution of the composites were determined to be less than 2 nm, which verified the micropores nature of both composites [41]. Figure 7 and the results obtained from Table 1 demonstrated that the metal oxide type could effectively control the morphological and textural properties of final ACNF composites, especially the pore system. 

**Table 1:** Textural characteristics of ACNF composites.ACNF/Fe2O3ACNF/Co3O4SBET (m2 g-1)212.21185.12Vtotal (cm3 g-1)0.1250.085Vmicro (cm³ g-1)0.0540.055Average pore diameter (nm)6.4775.741Average pore width (nm)2.3631.842

	ACNF/Fe2O3	ACNF/Co3O4
SBET (m2 g-1)	212.21	185.12
Vtotal (cm3 g-1)	0.125	0.085
Vmicro (cm³ g-1)	0.054	0.055
Average pore diameter (nm)	6.477	5.741
Average pore width (nm)	2.363	1.842

**Figure 8:**
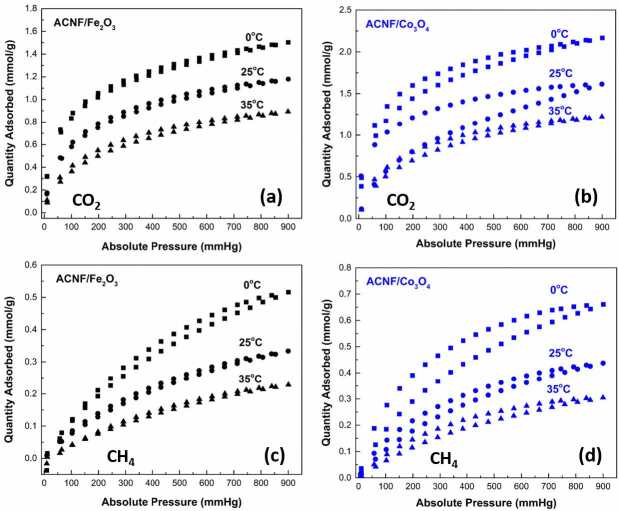
CO2 adsorption isotherms of (a) ACNF/Fe2O3 and (b) ACNF/Co3O4 composites.CH4 adsorption isotherms of (c) ACNF/Fe2O3 and (d) ACNF/Co3O4 composites.

The prepared ACNF composites were further used for adsorption studies of CO_2_ and CH_4_. Figure 8 (a)-(b) and (c)-(d) present adsorption-desorption isotherms of ACNFs determined up to 900 mmHg at 0, 25, and 35 °C for pure CO_2_ and CH_4_, respectively. The adsorption capacities obtained from these curves are summarized in Table 2. As expected, ACNF/Fe_2_O_3_ and ACNF/Co_3_O_4_ composites resulted the highest CO_2_ adsorption capacities of 1.502 and 2.166 mmol/g at 0 °C, respectively. Likewise, the highest CH_4_ adsorption capacities were obtained to be 0.516 and 0.661 mmol/g at 0 °C by ACNF/Fe_2_O_3_ and ACNF/Co_3_O_4_ composites, respectively. These differences could be attributed to their different average pore diameter. The fiber texture could lead to enhance the intraparticle adsorption kinetics in adsorption of gas molecules. Additionally, the presence of Co_3_O_4_ could provide stronger interactions with CO_2_ and CH_4_ that directly related to the increased chemisorption of gas molecules by the ACNFs [42]. Modulation of the lattice oxygen in metal oxides NPs and the and surface hydroxyl groups of ACNF composites could show a positive effect for the adsorption of CO_2_ and CH_4_ acting as acids [43]. Since these metal oxides in the ACNF composites are capable of donating an electrons pair without bonding, they are considered to be Lewis bases [44]. Therefore, the ACNF composites presented comparable CO_2_ and CH_4_ adsorption capacities. Zainab et al. prepared porous CNFs to develop efficient adsorbents for CO_2_ capture and reported that the prepared CNFs presented CO_2_ adsorption performance of 3.11 mmol/g [45]. In a different work, Abbasi et al. examined the amine-bearing nanofibrous adsorbent for the CO_2_ adsorption at ambient conditions [46]. They reported that the best CO_2_ adsorption capacity of 2.87 mmol/g was obtained after optimization. In a recent study, PAN/PVDF hybrid composites were prepared by electrospinning and used for CO_2_ capture after carbonization and activation process [47] the best CO_2_ uptake of 2.21 mmol/g under 1 bar pressure was obtained. 

**Table 2:** Maximum CO2 and CH4 adsorption capacities at 0 °C, 25 °C, and 35 °C up to 900 mmHg.

Adsorption capacity (mmoladsorbed/gadsorbent)	Adsorption temperature	ACNF/Fe2O3	ACNF/Co3O4
CO2	0 °C	1.502	2.166
CH4		0.516	0.661
CO2	25 °C	1.179	1.614
CH4		0.334	0.437
CO2	35 °C	0.893	1.223
CH4		0.228	0.305

Temperature also showed an important effect on the adsorption studies. When the temperature increased and absolute pressure decreased, both CO_2_ and CH_4_ adsorption capacities decreased, which indicates that the adsorption processes were exothermic through both composites. The exothermic adsorption process could occur owing to the gas molecules obtaining the required energy to overcome van der Waals forces and then returning back to gas phase [48].

In detail, it can be found that there was small hysteresis in both gases adsorption-desorption isotherms onto ACNF/Fe_2_O_3_. Contrary, the hysteresis became less pronounced for CO_2_ and CH_4_ adsorption-desorption isotherms onto ACNF/Co_3_O_4_, especially at low temperatures and low absolute pressure range. This hysteresis indicating the mesoporous structure of the ACNF composites, which matches with the findings in N_2_ adsorption-desorption experiments. As seen, CO_2_ and CH_4_ adsorption increased quickly with the increased absolute pressure due to the microporous structure [49]. It could be said that ACNF composites demonstrated multimodal pore size structure. 

The isosteric heats (Q_st_) of CO_2_ and CH_4_ adsorption for both composites were determined by applying Clausius–Clapeyron equation (Equation 1) to adsorption data at 0, 25, and 35°C in order to predict the type and strength of adsorption forces [50]. 

Q_st_=-R[(∂ lnP)/∂(1/T) ]_q_ (1)

By the integration of Equation 1, Equation 2 is obtained, as given:

(lnP )_q_=-(Q_st_/R)(1/T)+c (2)

where Q_st_ (kJ/mol) is the isosteric heat of adsorption and c is a constant. Q_st_ values were calculated from the slope of the curve obtained by plotting ln P vs. 1/T.

**Figure 9:**
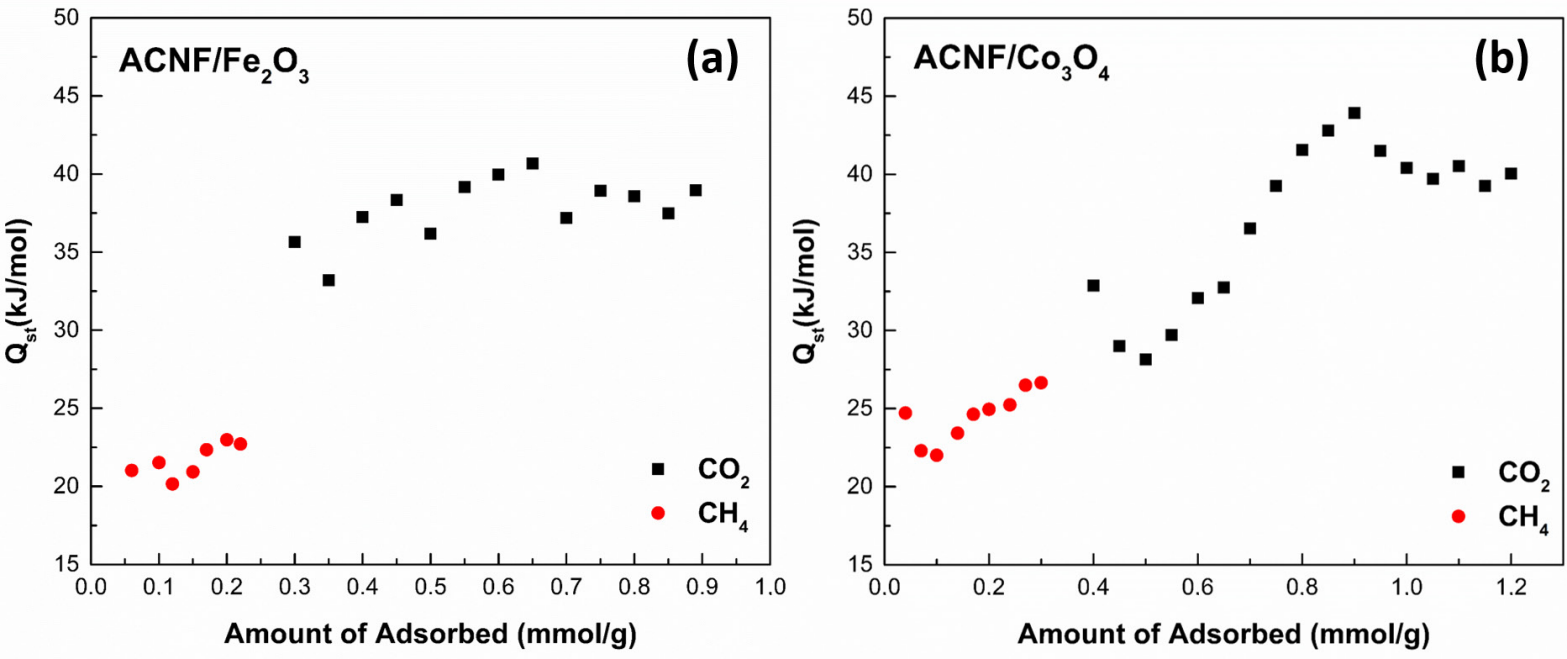
Isosteric heats of CO2 and CH4 adsorption as a function of the adsorbed amount for (a )ACNF/Fe2O3 and (b) ACNF/Co3O4 composites.

The Q_st_ values is considered to be a representative of the adsorption enthalpy change. Figure 9 (a) and (b) present the calculated Q_st _values as functions amount of adsorbed CO_2_ and CH_4_ by ACNF/Fe_2_O_3_ and ACNF/Co_3_O_4_ composites, respectively. As seen, all values increased with the increased amount of adsorbed gas molecules, in general. The Q_st _value of CO_2_ adsorption on ACNF/Fe_2_O_3_ was in the range of 33.2–40.7 kJ/mol, while it was in the range of 28.1–43.9 kJ/mol. These relatively higher Q_st _values could be related to the presence of micropores in both ACNF composites and a strong chemical interaction between metal oxides compounds and CO_2_ molecules [51]. The Q_st _values of CH_4_ adsorption on the ACNF/Fe_2_O_3_ and ACNF/Co_3_O_4_ composites were calculated in the range of 20.2–23.0 and 22.0–26.7 kJ/mol, respectively. These results were typical for porous adsorbents indicating that there was strong interaction between the CH_4 _molecules and pore walls of the ACNFs [50]. As a result, the Q_st_ values calculated lower than 80 kJ/mol showed that the adsorption processes of CO_2_ and CH_4_ were mainly dominated by physical adsorption agreeing with the findings of N_2_ adsorption-desorption experiments [52]. 

## 4. Conclusion

Herein, two different metal oxides influencing the morphological, structural, and textural properties have been identified. PAN based ACNF composites by incorporation of Fe_2_O_3_ and Co_3_O_4_ NPs were prepared via electrospinning, followed by stabilization, and thermal activation. The SEM images presented that the morphology of ACNF/Co_3_O_4_ composite fibers was more ordered and compact with an average diameter of 130 nm than that of ACNF/Fe_2_O_3_ composite fibers with an average diameter of 160 nm. S_BET_ values of ACNF/Fe_2_O_3_ and ACNF/Co_3_O_4_ composites were determined to be 212.21 and 185.12 m^2^/g, respectively, which makes the composites available for gas adsorption process. The ACNF composites were utilized as adsorbents for CO_2_ and CH_4_ adsorption. Comparing the adsorption capacities indicated that the ACNF/Co_3_O_4_ composite showed higher capacity than ACNF/Fe_2_O_3_ composite, which could be related to chemical interaction between Co_3_O_4_ NPs and gas molecules. Temperature depending adsorption studies revealed that the lower temperatures were more suitable for adsorption studies by both composites. In summary, the results obtained indicated that ACNF-metal oxide composites are good candidates for designing of efficient CO_2_ and CH_4_ adsorption systems.
